# Estimation of the effect of dalfampridine-ER on health utility by mapping the MSWS-12 to the EQ-5D in multiple sclerosis patients

**DOI:** 10.1186/1477-7525-11-105

**Published:** 2013-06-26

**Authors:** Brendan L Limone, Matthew F Sidovar, Craig I Coleman

**Affiliations:** 1 University of Connecticut School of Pharmacy, Storrs, CT, USA; 2 Acorda Therapeutics, Ardsley, NY, USA; 3 University of Connecticut School of Pharmacy, 80 Seymour Street, Hartford, CT 06102, USA

**Keywords:** Dalfampridine, EQ-5D, MSWS-12, Multiple sclerosis

## Abstract

**Background:**

Trials have not assessed the effect of dalfampridine-extended release (dalfampridine-ER) on health utility. We sought to evaluate the effect of dalfampridine-ER tablets (prolonged-release fampridine in Europe) on health utility in patients with multiple sclerosis (MS) by mapping subjects’ individual item scores from the 12-Item Multiple Sclerosis Walking Scale (MSWS-12) onto the Euroqol 5-Dimension (EQ-5D) health utility index.

**Methods:**

Data from study MS-F203, a randomized trial of dalfampridine-ER tablets, 10 mg twice daily, in patients with MS, were used to calculate the health utility scores with two MSWS-12 to EQ-5D mapping equations (one derived in a North American [NA] registry, the other a United Kingdom [UK] registry). MS-F203 participants were categorized as dalfampridine-ER 20%-responders (achieving ≥20% improvement on the Timed 25-Foot Walk), dalfampridine-ER 20%-nonresponders (<20% improvement), or placebo patients. Mean change in health utility scores from baseline to each double-blind treatment evaluation (visits 3-6 occurring at post-randomization weeks 2, 6, 10, and 14) and each off-drug follow-up evaluation (visits 7-8 occurring at weeks 16 and 18) were calculated and reported as effect sizes (ESs).

**Results:**

Using the NA-derived equation, dalfampridine-ER 20%-responders demonstrated improvement in health utility vs. placebo; starting at week 6 (mean difference in ES = 0.44, p = 0.002) and maintained at weeks 10 (ES = 0.41, p = 0.01) and 14 (ES = 0.71, p < 0.001). These improvements were no longer evident after dalfampridine-ER was discontinued (p > 0.05 at weeks 16 and 18). Dalfampridine-ER 20%-nonresponders did not show improvement vs. placebo at any visit (p > 0.05 for all). When using the UK-derived equation, improvement was seen in dalfampridine-ER 20%-responders vs. placebo at weeks 2, 6, 10, and 14 (ESs = 0.49, 0.55, 0.59, and 0.99; p < 0.03 for all), but not when dalfampridine-ER was discontinued (weeks 16 and 18; p > 0.05 for both). Dalfampridine-ER 20%-nonresponders showed no improvement at any visit (p > 0.05 for all).

**Conclusion:**

Regardless of the equation used, dalfampridine-ER response was associated with an improvement in health utility.

## Introduction

Multiple Sclerosis (MS), a chronic and progressive neurologic disease affecting approximately 400,000 Americans, is primarily diagnosed between the ages of 18 and 45 years [1]. Mobility impairment is a major concern of MS patients, and data suggest that even mild mobility loss associated with MS may adversely affect health-related quality of life (HrQoL) [2,3].

The 12-Item Multiple Sclerosis Walking Scale (MSWS-12) is a validated rating scale that captures patients’ perceptions of the impact of MS on walking ability [4]. The MSWS-12 was used as to validate the clinical significance of changes in walking speed (as measured by the Timed 25-Foot Walk [T25FW]) in a Phase 3 randomized, controlled trial of dalfampridine extended release tablets (dalfampridine-ER; known as prolonged-release fampridine in Europe and as sustained or modified release fampridine elsewhere) in MS patients (MS-F203). Dalfampridine-ER is an oral, broad spectrum potassium channel blocker that is indicated to improve walking in patients with MS. In this study, mean changes from baseline MSWS-12 score on a 100 point scale during the 14-week double-blind treatment period, independent of treatment assignment, were -6.84 (95% confidence interval [CI]: -9.65 to -4.02) for Timed 25-Foot Walk (T25FW) responders (using the trial’s own definition of response) and 0.05 (95%CI: -1.48 to 1.57) for non-responders, (p = 0.0002), indicating a reduction in patient-assessed walking impairment in T25FW responders. This trial also demonstrated that dalfampridine-ER produced clinically meaningful improvement in walking speed in a proportion of people with MS as measured by the T25FW test, regardless of disease course or concomitant use of disease modifying agents [5]. Despite the fact that dalfampridine-ER is a drug to treat a symptom of MS (walking impairment) highly associated with decreased HrQoL, no data assessment of the effect of dalfampridine-ER on a measure of health utility or HrQoL has been published. The availability of such data would aid decision- and policy-makers in making coverage decisions and investigators in future research endeavors.

Two groups have independently developed equations to map the MSWS-12 onto the Euroqol 5-Dimension (EQ-5D) [6], a validated, generic, preference-based health status measure. Both equations utilize the responses from individual items of the MSWS-12 to derive health utility scores (measures of HrQoL); however, one equation was derived in a large North American (NA) population [7] and the other in a smaller United Kingdom (UK) population [8]. As no one equation perfectly fits any population, we used both of the aforementioned MSWS-12 to EQ-5D mapping equations to address our primary objective which was to evaluate the effect of dalfampridine-ER on health utility and HrQoL.

## Methods

Data for these post-hoc analyses were taken from study MS-F203 (clinicaltrials.gov registration: NCT00127530), a Phase 3, randomized, double-blind, parallel group, placebo-controlled, multi-center clinical trial conducted in the US and Canada. The objective was to assess the efficacy and safety of dalfampridine-ER to improve walking in patients with MS. The primary outcome measure in this trial was improvement in walking speed, in feet per second, as measured by the T25FW test during the double-blind treatment period. Patients were screened prior to receiving study medication; eligible patients (those aged 18–70 years with clinically defined MS, who were able to complete two trials of the T25FW in an average time of 8–45 seconds) returned one week later (visit 0). Patients then entered a two-week, single-blind, placebo run-in period (visit 1 at the beginning of week 2 of placebo run-in). At the end of the placebo run-in (visit 2), patients were randomly assigned to receive dalfampridine-ER at a dose of 10 mg twice daily or placebo. After two weeks, patients returned for the first double-blind assessments (visit 3). Thereafter, patients returned every four weeks for a total of 14 weeks of double-blind treatment (visits 4, 5, and 6 corresponding to weeks 6, 10, and 14). At the end of the 14-week double-blind treatment period, patients began a four-week period of no treatment, returning for follow-up assessments at two-week intervals (visits 7 and 8 corresponding to weeks 16 and 18) [5].

A secondary outcome measure in study MS-F203 was the MSWS-12. The MSWS-12 is a validated, patient-reported functional outcome measure assessing patients’ perceptions of the impact of MS on their walking ability. The MSWS-12 includes 12 questions that are rated on a scale ranging between 1 (“Not at all”) and 5 (“Extremely”). Each of the 12 questions asks about a different aspect of walking, such as ability and speed of walking; ability to run; ability to climb and descend stairs; balance and smoothness of gait; support, effort, and concentration required. Total scores are calculated and range from 12–60, or, to aid in interpretation, can be reported as transformed scores ranging from 0–100. Higher scores reflect a greater negative impact on walking ability [4]. In study MS-F203, the MSWS-12 was assessed at visits 0, 2, 3, 4, 5, 6, 7, and 8 [5].

Two unique and independently derived mapping equations were used to cross-walk MSWS-12 individual item scores from study MS-F203 to health utility scores using the EQ-5D. The first equation was derived using over 3,500 participants (mean EQ-5D and MSWS-12 scores were 0.74 ± 0.18 and 50.8 ± 33.5, respectively) in the North American Research Committee on Multiple Sclerosis (NARCOMS) registry using ordinary least squares (OLS) regression and scoring the EQ-5D using the US scoring algorithm (which ranges from -0.11 to 1.0 on a scale where 0.0 = death and 1.0 = perfect health). The equation (EQ-5D_US_ = 0.002* [Item 1] -0.009* [Item 2] -0.01* [Item 3] -0.029* [Item 4] -0.019* [Item 5] -0.0000881* [Item 6] -0.008* [Item 7] -0.002* [Item 8] +0.013* [Item 9] -0.011* [Item 10] +0.001* [Item 11] -0.008* [Item 12] +0.983) uses all of the individual MSWS-12 item scores to estimate EQ-5D index scores with a mean absolute error (MAE) of 0.109 ± 0.096 [7]. Hawton and colleagues used data from 560 people with MS in South West England (SWIMS project; mean EQ-5D and MSWS-12 scores were 0.61 ± 0.25 and 60.1 ± 32.4, respectively), and similarly to the abovementioned equation, found the OLS regression using all individual MSWS-12 item scores (except for item 11, which was removed because of colinearity) to be the best performing equation for calculating EQ-5D using the UK scoring algorithm (which ranges from -0.594 to 1.0 on a scale where 0.0 = death and 1.0 = perfect health), with a MAE = 0.148, 95% confidence interval, 0.138 to 0.159 (EQ-5D_UK_ = -0.0004282* [Item 1] -0.0029117* [Item 2] -0.0213846* [Item 3] -0.0410001* [Item 4] +0.0086472* [Item 5] -0.0340533* [Item 6] -0.0154952* [Item 7] -0.0180406* [Item 8] +0.0004532* [Item 9] +0.0148398* [Item 10] -0.004274* [Item 12] +0.9843433) [8].

Analyses included all patients from the study MS-F203 modified intent-to-treat (mITT) population (all randomized patients who received double-blind investigational drug and who had at least one subsequent primary efficacy assessment during the double-blind treatment period) who had complete MSWS-12 data from at least one baseline and one double-blind or follow-up visit [5]. We estimated mean change in health utility score from baseline for each patient using the mapping equations and reported the results as effect sizes (ESs) by calculating change in health utility score and dividing these by the standard deviation (SD) of health utility score of all patients at baseline.

The *a priori* definition of T25FW response in the MS-F203 trial was the occurrence of faster walking speed for at least three of the four visits during the double-blind treatment period compared to the maximum speed for any of the first five off-drug visits. However, previous reports have suggested that a 20% improvement in T25FW represents the minimally important clinical difference, or the smallest difference in an outcome measure that is perceived as beneficial by the patient [9-11]. Therefore, we utilized this latter definition for this analysis and categorized patients as a 20%-responder or 20%-nonresponder based on whether their mean of all T25FW assessments during double-blind visits improved by 20% or more from the mean of the pre-randomization visits baseline. Differences in mean ESs were then compared between 20%-responders or 20%-nonresponders, and for dalfampridine-ER 20%-responders (randomized to receive dalfampridine-ER and achieved a ≥20% improvement), dalfampridine-ER 20%-nonresponders and placebo patients. ES has been recommended in the literature as an appropriate benchmark for evaluating the magnitude and clinically-relevant meaning of change in an endpoint measure, with ESs of ≥0.2, ≥0.5, and ≥0.8 interpreted as small, medium, and large, respectively [11-14].

Categorical data were compared using chi-squared tests. Continuous data were compared using either an unpaired *t*-test or one-way analysis of variance (ANOVA) test with Bonferroni post-hoc t-tests, where appropriate. A p-value <0.05 was considered statistically significant in all situations. All analyses were conducted using SPSS version 17.0 (SPSS Inc., Chicago, IL, USA).

## Results

In study MS-F203, a total of 301 patients were randomized, but one failed to take any double-blind treatment and was thus excluded. Of the remaining 300, an additional 4 withdrew from the study before completing any double-blind assessments, for a total of 296 patients in the mITT population. As depicted in Table 1, the two treatment groups were comparable in demographic characteristics at baseline. Of these patients, 293 had at least one pre-randomization MSWS-12 assessment, an inclusion criterion specific to this analysis. Of these, 221 received dalfampridine-ER, of which 73 (33%) were categorized as 20%-responders, and 72 (33%) received placebo. The mean MSWS-12 ± SE scores at baseline for dalfampridine-ER 20%-responders, dalfampridine-ER 20%-nonresponders and placebo were 66.2 ± 2.32, 70.7 ± 1.69, and 67.4 ± 2.78, respectively. Mean EQ-5D ± SE scores calculated for these groups using the NA-derived equation were 0.70 ± 0.006, 0.69 ± 0.005, and 0.70 ± 0.008, respectively. No significant differences in MSWS-12 or EQ-5D scores were observed between groups at baseline.

**Table 1:** Baseline characteristics of the modified intention-to-treat population from study MS-F203

**Characteristic**	**All patients**	**Placebo**	**Dalfampridine-ER 10 mg BID**	**P-value**
	**(N = 296)**	**(N = 72)**	**(N = 224)**	
Age, years (mean ± SE)	51.4 ± 0.51	50.9 ± 1.05	51.5 ± 0.58	0.50
Female gender, n (%)	201 (67.9)	43 (59.7)	158 (70.5)	0.11
MS Diagnosis Type				0.52
Primary progressive	44 (14.9)	14 (19.4)	30 (13.4)	
Progressive relapsing	12 (4.1)	2 (2.8)	10 (4.5)	
Relapsing remitting	82 (27.7)	21 (29.2)	61 (27.2)	
Secondary progressive	158 (53.4)	35 (48.6)	123 (54.9)	
Disease Duration, years (mean ± SE)	13.4 ± 0.48	12.7 ± 0.97	13.6 ± 0.55	0.38
EDSS score (mean ± SE)	5.8 ± 0.06	5.8 ± 0.13	5.8 ± 0.07	0.85
Range (Minimum, Maximum)	(3, 7)	(3, 7)	(3, 7)	
Timed 25-Foot Walk speed, feet/second (mean ± SE)	2.1 ± 0.04	2.1 ± 0.08	2.1 ± 0.05	0.85

*BID* twice daily, *EDSS* Expanded Disability Status Scale, *ER* extended-release, *MS* multiple sclerosis, *SE* standard error.

### NA-derived equation results

**Figure 1:**
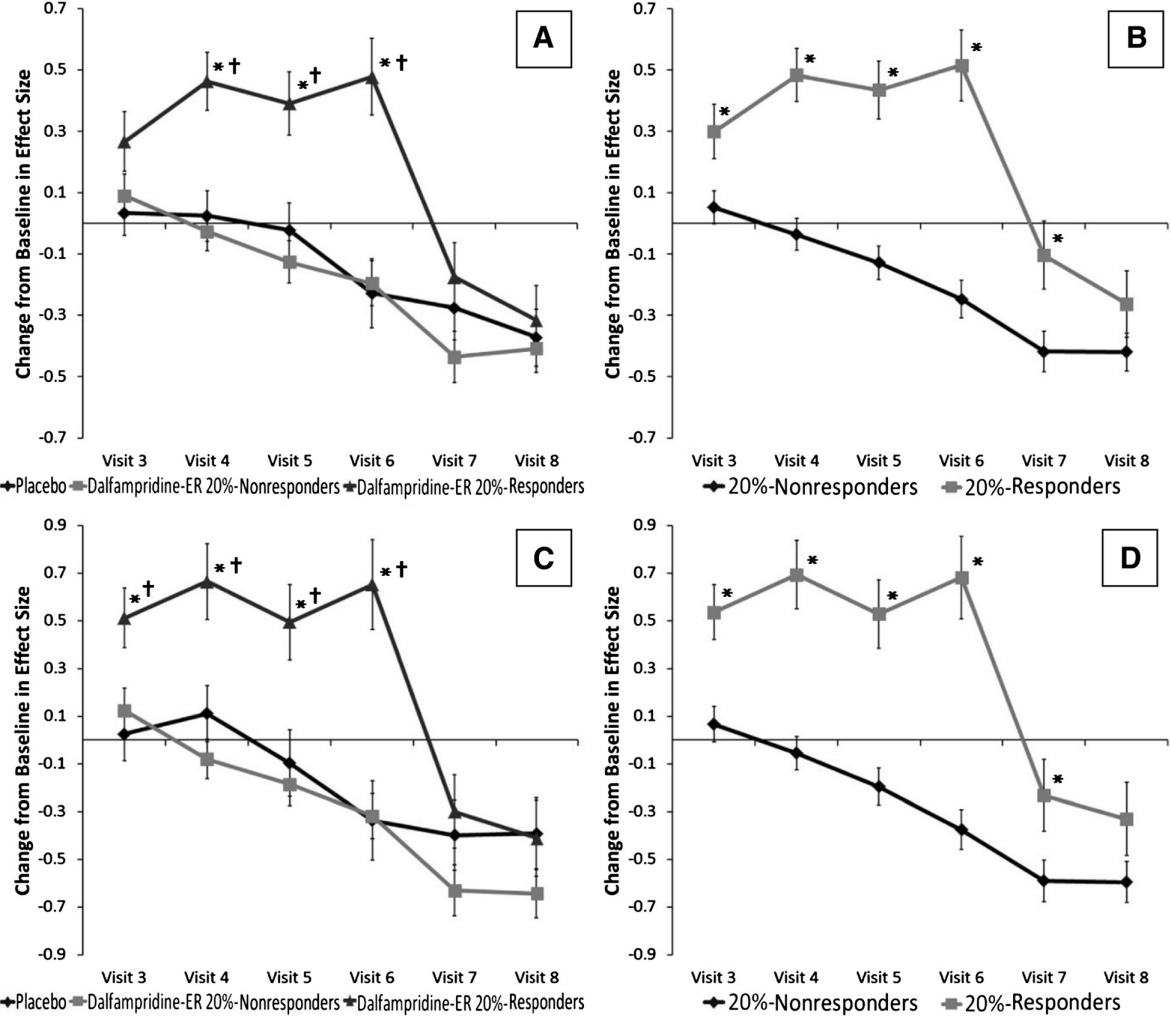
**Effect sizes of change in EQ-5D by responder status and treatment group.** Change in effect size for EQ-5D when stratified by treatment group (dalfampridine-ER 20%-responder status or placebo) and 20%-responder status (≥20% percent increase in Timed 25-Foot Walk speed or < 20%) during on-treatment visits 3-6 and off treatment visits 7/8. **A**, **B**) results using North American-derived equation; **C**, **D**) results using United Kingdom-derived equation. *p < 0.05 versus D-ER nonresponders; †p < 0.05 versus placebo.

Using the NA-derived equation, dalfampridine-ER 20%-responders exhibited small improvements in health utility vs. placebo starting at week six (visit 4; mean difference in change score between groups: 0.028; mean difference in ES between groups: 0.44, p = 0.002). (Table 2) These improvements were maintained at week 10 (visit 5; 0.026; 0.41, p = 0.009) and increased to moderate at week 14 (visit 6; 0.044; 0.71, p < 0.001). After treatment discontinuation, improvements in health utility were no longer apparent at week 16 (visit 7; 0.006; 0.10, p = NS) or week 18 (visit 8; 0.004, 0.06, p = NS). Similarly, dalfampridine-ER 20%-responders demonstrated small improvements in health utility vs. dalfampridine-ER 20%-nonresponders starting at week 6 (visit 4; 0.031; 0.49, p < 0.001). These increased to moderate improvements at week 10 (visit 5; 0.032; 0.52, p < 0.001) and were maintained at week 14 (visit 6; 0.042; 0.67, p < 0.001). No improvements were apparent at either week 16 (visit 7; 0.016; 0.26, p = NS) or week 18 (visit 8; 0.006; 0.092, p = NS) after treatment discontinuation. No significant differences were observed between dalfampridine-ER 20%-nonresponders and placebo at any visit (p > 0.05 for all) (Figure 1A).

**Table 2:** Comparison of dalfampridine-ER 20%-nonresponders, 20%-responders and placebo using the North American-derived equation

	**Placebo**	**Dalfampridine-ER 20%-nonresponders**^**a**^	**Dalfampridine-ER 20%-responders**^**a**^	**P-value DAL-R vs. placebo**	**P-value DAL-R vs. DAL-NR**
	**Mean ± SE**	**Mean ± SE**	**Mean ± SE**		
Average of placebo run-in and randomization (Visits 0, 1, 2)	n = 72	n = 148	n = 73		
EQ-5D at baseline	0.696 ± 0.008	0.691 ± 0.005	0.699 ± 0.006	NS	NS
Week 2 (Visit 3)	n = 70	n = 145	n = 71		
Change from baseline, EQ-5D	0.002 ± 0.005	0.006 ± 0.004	0.017 ± 0.006	NS	NS
Change from baseline, ES	0.033 ± 0.072	0.090 ± 0.069	0.267 ± 0.097		
Week 6 (Visit 4)	n = 70	n = 144	n = 71		
Change from baseline, EQ-5D	0.002 ± 0.005	−0.002 ± 0.004	0.029 ± 0.006	0.002	<0.001
Change from baseline, ES	0.024 ± 0.082	−0.027 ± 0.063	0.464 ± 0.94		
Week 10 (Visit 5)	n = 68	n = 142	n = 68		
Change from baseline, EQ-5D	−0.001 ± 0.006	−0.008 ± 0.004	0.025 ± 0.006	0.009	<0.001
Change from baseline, ES	−0.022 ± 0.089	−0.126 ± 0.069	0.391 ± 0.102		
Week 14 (Visit 6)	n = 70	n = 139	n = 70		
Change from baseline, EQ-5D	−0.014 ± 0.007	−0.012 ± 0.005	0.030 ± 0.008	<0.001	<0.001
Change from baseline, ES	−0.227 ± 0.113	−0.196 ± 0.074	0.478 ± 0.124		
Week 16 (Visit 7)	n = 71	n = 144	n = 71		
Change from baseline, EQ-5D	−0.017 ± 0.007	−0.027 ± 0.005	−0.011 ± 0.007	NS	NS
Change from baseline, ES	−0.276 ± 0.105	−0.436 ± 0.083	−0.176 ± 0.113		
Week 18 (Visit 8)	n = 70	n = 140	n = 72		
Change from baseline, EQ-5D	−0.023 ± 0.006	−0.026 ± 0.005	−0.020 ± 0.007	NS	NS
Change from baseline, ES	−0.373 ± 0.092	−0.409 ± 0.078	−0.376 ± 0.053		

*DAL* dalfampridine, *EQ-5D* Euroqol 5-Dimension, *ES* effect size, *NR* nonresponder, *NS* not significant, *R* responder, *SE* standard error.

See Figure 1A for graphical representation.

^**a**^20%-nonresponder or 20%-responder status based on whether the mean of all T25FW scores during double-blind visits improved by 20% or more from the mean of the T25FW scores during pre-randomization visits.

With the NA-derived equation, 20%-responders demonstrated small improvements in health utility vs. 20%-nonresponders starting at week two (visit 3; mean difference in change score between groups: 0.016; mean difference in ES between groups: 0.25, p = 0.016). (Table 3) Moderate improvements were maintained at week six (visit 4; 0.033; 0.52, p < 0.001), week 10 (visit 5; 0.035; 0.56, p < 0.001), and week 14 (visit 6; 0.048; 0.76, p < 0.001). These improvements began to decline after treatment discontinuation at week 16 (visit 7; 0.020; 0.31, p = 0.013) and by week 18, significant improvements in health utility no longer existed (visit 8; 0.010; 0.16, p = 0.187) (Figure 1B).

**Table 3:** Comparison of 20%-nonresponders vs. 20%-responders using the North American-derived equation

	**20%-nonresponders**^**a**^	**20%-responders**^**a**^	**P-value**
	**Mean ± SE**	**Mean ± SE**	
Average of placebo run-in and randomization (Visits 0, 1, 2)	n = 212	n = 81	
EQ-5D at baseline	0.693 ± 0.005	0.698 ± 0.006	NS
Week 2 (Visit 3)	n = 207	n = 79	
Change from baseline, EQ-5D	0.003 ± 0.003	0.019 ± 0.006	0.02
Change from baseline, ES	0.052 ± 0.054	0.300 ± 0.088	
Week 6 (Visit 4)	n = 206	n = 79	
Change from baseline, EQ-5D	−0.002 ± 0.003	0.030 ± 0.005	<0.001
Change from baseline, ES	−0.036 ± 0.051	0.483 ± 0.086	
Week 10 (Visit 5)	n = 202	n = 76	
Change from baseline, EQ-5D	−0.008 ± 0.004	0.027 ± 0.006	<0.001
Change from baseline, ES	−0.128 ± 0.055	0.435 ± 0.094	
Week 14 (Visit 6)	n = 201	n = 78	
Change from baseline, EQ-5D	−0.016 ± 0.004	0.032 ± 0.007	<0.001
Change from baseline, ES	−0.248 ± 0.062	0.515 ± 0.115	
Week 16 (Visit 7)	n = 207	n = 79	
Change from baseline, EQ-5D	−0.026 ± 0.004	−0.007 ± 0.007	0.01
Change from baseline, ES	−0.418 ± 0.065	−0.104 ± 0.110	
Week 18 (Visit 8)	n = 203	n = 79	
Change from baseline, EQ-5D	−0.026 ± 0.004	−0.016 ± 0.007	NS
Change from baseline, ES	−0.420 ± 0.061	−0.263 ± 0.108	

*EQ-5D* Euroqol 5-Dimension, *ES* effect size, *NS* not significant *SE* standard error, *T25FW* Time 25 Foot Walk.

See Figure 1B for graphical representation.

^**a**^20%-nonresponder or 20%-responder status based on whether the mean of all T25FW scores during double-blind visits improved by 20% or more from the mean of the T25FW scores during pre-randomization visits.

### UK-derived equation results

Using the UK-derived equation, dalfampridine-ER 20%-responders demonstrated small improvements in health utility vs. placebo starting at week two (visit 3; mean difference in change score between groups: 0.031; mean difference in ES between groups: 0.49, p = 0.022). (Table 4) Moderate improvements were apparent by week six (visit 4; 0.035; 0.55, p = 0.009), maintained through week 10 (visit 5; 0.037; 0.59, p = 0.009), and increased to large improvements by week 14 (visit 6; 0.062; 0.99, p < 0.001). After treatment discontinuation, improvements were no longer significant at week 16 (visit 7; 0.006; 0.010, p = NS) or week 18 (visit 8; -0.001; -0.020, p = NS). Dalfampridine-ER 20%-responders demonstrated small improvements in health utility vs. dalfampridine-ER 20%-nonresponders starting at week two (visit 3; 0.24; 0.39, p = 0.039). Moderate improvements were observed at week six (visit 4; 0.47; 0.74, p < 0.001), maintained at week 10 (visit 5; 0.43; 0.68, p < 0.001), and increased to large improvements at week 14 (visit 6; 0.61; 0.97, p < 0.001). Significant improvements were not seen at week 16 (visit 7; 0.021; 0.33, p = NS) or week 18 (visit 8; 0.015; 0.23, p = NS) after discontinuation of treatment. Dalfampridine-ER 20%-nonresponders did not show improvements in health utility vs. placebo at any visit (p > 0.05 for all) (Figure 1C).

**Table 4:** Comparison of dalfampridine- ER 20%-nonresponders, 20%-responders and placebo using the United Kingdom-derived equation

	**Placebo**	**Dalfampridine-ER 20%-nonresponders**^**a**^	**Dalfampridine-ER 20%-responders**^**a**^	**P-value DAL-R vs. placebo**	**P-value DAL-R vs. DAL-NR**
	**Mean ± SE**	**Mean ± SE**	**Mean ± SE**		
Average of placebo run-in and randomization (Visits 0, 1, 2)	n = 72	n = 148	n = 73		
EQ-5D at baseline	0.574 ± 0.012	0.568 ± 0.008	0.579 ± 0.083	NS	NS
Week 2 (Visit 3)	n = 70	n = 145	n = 71		
Change from baseline, EQ-5D	0.002 ± 0.007	0.008 ± 0.006	0.032 ± 0.008	0.022	0.04
Change from baseline, ES	0.025 ± 0.111	0.124 ± 0.094	0.512 ± 0.126		
Week 6 (Visit 4)	n = 70	n = 144	n = 71		
Change from baseline, EQ-5D	0.007 ± 0.008	−0.005 ± 0.005	0.042 ± 0.085	0.009	<0.001
Change from baseline, ES	0.111 ± 0.119	−0.080 ± 0.084	0.665 ± 0.160		
Week 10 (Visit 5)	n = 68	n = 142	n = 68		
Change from baseline, EQ-5D	−0.006 ± 0.009	−0.012 ± 0.006	0.031 ± 0.010	0.009	<0.001
Change from baseline, ES	−0.097 ± 0.140	−0.185 ± 0.089	0.495 ± 0.158		
Week 14 (Visit 6)	n = 70	n = 139	n = 70		
Change from baseline, EQ-5D	−0.021 ± 0.010	−0.020 ± 0.006	0.041 ± 0.012	<0.001	<0.001
Change from baseline, ES	−0.337 ± 0.167	−0.319 ± 0.095	0.652 ± 0.188		
Week 16 (Visit 7)	n = 71	n = 144	n = 71		
Change from baseline, EQ-5D	−0.025 ± 0.009	−0.040 ± 0.007	−0.019 ± 0.010	NS	NS
Change from baseline, ES	−0.399 ± 0.148	−0.631 ± 0.107	−0.300 ± 0.155		
Week 18 (Visit 8)	n = 70	n = 140	n = 72		
Change from baseline, EQ-5D	−0.025 ± 0.010	−0.041 ± 0.006	−0.026 ± 0.010	NS	NS
Change from baseline, ES	−0.392 ± 0.151	−0.643 ± 0.103	−0.411 ± 0.160		

*DAL* dalfampridine, *EQ-5D* Euroqol 5-Dimension, *ES* effect size, *NR* nonresponder, *NS* not significant, *R* responder, *SE* standard error.

See Figure 1C for graphical representation.

^**a**^20%-nonresponder or 20%-responder status based on whether the mean of all T25FW scores during double-blind visits improved by 20% or more from the mean of the T25FW scores during pre-randomization visits.

Simlarly, 20%-responders demonstrated small improvements in health utility vs. 20%-nonresponders starting at week two (visit 3; mean difference in change score between groups: 0.030; mean difference in ES between groups: 0.47, p = 0.001). (Table 5) Moderate improvements were observed at week six (visit 4; 0.047; 0.75, p < 0.001), maintained at week 10 (visit 5; 0.046; 0.72, p < 0.001), and increased to large improvements by week 14 (visit 6; 0.067; 1.06, p < 0.001). After treatment discontinuation, these improvements began to decline at week 16 (visit 7; 0.022; 0.36, p = 0.034) and no significant improvement was seen by week 18 (visit 8; 0.017; 0.26, p = NS) (Figure 1D).

**Table 5:** Comparison of 20%-nonresponders vs. 20%-responders using the United Kingdom-derived equation

	**20%-nonresponders**^**a**^	**20%-responders**^**a**^	**P-value**
	**Mean ± SE**	**Mean ± SE**	
Average of placebo run-in and randomization (Visits 0, 1, 2)	n = 212	n = 81	
EQ-5D at baseline	0.570 ± 0.007	0.577 ± 0.009	NS
Week 2 (Visit 3)	n = 207	n = 79	
Change from baseline, EQ-5D	0.004 ± 0.005	0.034 ± 0.007	0.001
Change from baseline, ES	0.067 ± 0.075	0.535 ± 0.115	
Week 6 (Visit 4)	n = 206	n = 79	
Change from baseline, EQ-5D	−0.004 ± 0.004	0.044 ± 0.009	<0.001
Change from baseline, ES	−0.055 ± 0.070	0.694 ± 0.144	
Week 10 (Visit 5)	n = 202	n = 76	
Change from baseline, EQ-5D	−0.012 ± 0.005	0.033 ± 0.009	<0.001
Change from baseline, ES	−0.195 ± 0.076	0.529 ± 0.144	
Week 14 (Visit 6)	n = 201	n = 78	
Change from baseline, EQ-5D	−0.024 ± 0.005	0.043 ± 0.011	<0.001
Change from baseline, ES	−0.376 ± 0.084	0.682 ± 0.173	
Week 16 (Visit 7)	n = 207	n = 79	
Change from baseline, EQ-5D	−0.037 ± 0.006	−0.015 ± 0.010	0.03
Change from baseline, ES	−0.590 ± 0.087	−0.232 ± 0.150	
Week 18 (Visit 8)	n = 203	n = 79	
Change from baseline, EQ-5D	−0.038 ± 0.005	−0.021 ± 0.010	NS
Change from baseline, ES	−0.596 ± 0.086	−0.331 ± 0.153	

*EQ-5D* Euroqol 5-Dimension, *ES* effect size, *NS* not significant, *SE* standard error, *T25FW* Timed 25 Foot Walk.

See Figure 1D for graphical representation.

^**a**^20%-nonresponder or 20%-responder status based on whether the mean of all T25FW scores during double-blind visits improved by 20% or more from the mean of the T25FW scores during pre-randomization visits.

## Discussion

The Data from this post-hoc secondary analysis of the double-blind, placebo-controlled clinical study MS-F203 demonstrate that dalfampridine-ER responders experienced a small-to-moderate, but clinically-relevant improvement in health utility starting six weeks after initiation of double-blind treatment, regardless of the mapping equation used. These results are further supported by our additional analyses showing 20%-responders performed better than non-responders, regardless of treatment assignment. Furthermore, our analysis suggests that these improvements in health utility were consistently reported by dalfampridine-ER responders for an additional 8 weeks while on treatment; however, once double-blind treatment was stopped, benefits began to dissipate.

The pattern of effect on health utility seen for dalfampridine-ER 20%-responders, dalfampridine-ER 20%-nonresponders, and placebo patients in our analysis generally mimicked that seen in study MS-F203 for the primary study endpoint of T25FW speed. In study MS-F203, patients deemed T25FW responders to dalfampridine (defined as a patient with a faster walking speed for at least three of the four visits during the double-blind treatment period than the maximum speed for any of the first five off-drug visits) showed a sustained improvement in T25FW speed during the treatment period starting at week two, which was completely reversed at the two-week and four-week off-drug follow-up visits [5]. Improvements were greater in dalfampridine-ER T25FW responders than in placebo and dalfampridine-ER T25FW nonresponders at all on-drug visits (p < 0.001 for all). Of note, we observed a one-visit (up to four-week) delay in achieving statistically significant improvements in health utility compared to T25FW speed in study MS-F203. This may suggest that patients’ perceived improvement in HrQoL lags somewhat behind actual improvements in walking speed, or may simply represent a statistical anomaly. Regardless, the similarities between our health utility analysis and the T25FW data provide some added validation of our results, particularly when one keeps in mind that T25FW is an unique measure of mobility from the MSWS-12 from which our EQ-5D scores were derived.

While the aim of this analysis was not to compare the results of the NA- and UK-derived equations, our analysis did demonstrate they yielded somewhat similar health utility when we applied to data from study MS-F203. That being said, important differences in these equations’ initial derivation databases may partially explain the differences in comparative effect estimates seen in our analysis with these two equations. The NA mapping equation was derived using data from a large cohort of NA (US and Canada) patients (n = 3,505) included in the NARCOMS database; the UK equation from Hawton and colleagues was derived in a significantly smaller cohort of MS patients in South West England (SWIMS project, n = 560). Moreover, the UK–based SWIMS database not surprisingly or inappropriately utilized the UK scoring system for the EQ-5D, which has a much wider range of health state values (-0.594 for the worst possible state to 1.00 for the best) than the US scoring algorithm used by NARCOMS (-0.109 for the worst health state to 1.00 for the best) [15]. According to guidance on health state utility mapping from the National Institute for Health and Clinical Excellence’s (NICE’s) it is important there be similarity between characteristics of the estimation and target samples in order for the findings of any future analysis to be valid [16]. The NA-derived equation not only used MS patients from the same geographic region as MS-F203, North America, but was also shown to be most precise (MAE of 0.087 ± 0.061) in patients with a Patient-Determined Disease Steps (PDDS) between 3 and 6 (correlating with an EDSS score of 4-6.5), the same degree of mobility impairment seen in MS-F203 (mean EDSS of 5.8) [3]. Thus, it would seem most appropriate to put somewhat greater weight on health utility values and effect sizes for study MS-F203 stemming from the NA-derived equation. Though beyond the scope of this analysis, future research into the cost-effectiveness of dalfampridine-ER can now be accomplished by utilizing the results of this analysis. It is important to note, health utility values reported here should be carefully selected based upon country perspective of future cost-effectiveness analyses. There are some limitations to our analysis worth further discussion. First, as highlighted in the NICE Decision Support Unit guidance, mapping is “at best, a second-best solution” to the direct collection of EQ-5D values [16]. However, as no health utility values were collected as part of the two Phase 3 clinical trials of dalfampridine-ER, our data represents the best estimates of the drug’s effect on health utility currently available. Moreover, since no mapping equation is perfect, we used both available equations to map the MSWS-12 to the EQ-5D available at the time. The fact that when both equations were applied to dalfampridine-ER clinical trial data yield similar results lends credence to our overall conclusion that dalfampridine-ER responders realize an improvement in health utility. Next, the post-hoc nature of data analysis from study MS-F203 lends itself to the possibility of type two error in our results. As this was not the primary, or even a prospectively defined, analysis of data from study MS-F203, inadequate sample sizes may have precluded us from showing clinically important differences at all time points.

## Conclusion

Regardless of the mapping equation used, results suggest dalfampridine-ER response is associated with a noteworthy improvement in health utility in MS patients. The UK-derived equation resulted in larger estimates of improvement than the NA-derived equation. The results presented in the current analysis may aid decision- and policy-makers in coverage decisions and aid investigators in designing and conducting future research into the clinical- and cost-effectiveness of dalfampridine-ER.

### Consent

Written informed consent was obtained from all patients for the publication of this report and any accompanying images.

## Competing interests

Dr. Coleman has received grant funding from Acorda Therapeutics, Mr. Sidovar is a paid employee of Acorda Therapeutics, Dr. Limone has no conflicts of interest to disclose.

## Authors’ contributions

CIC and MFS had full access to all of the data in the study and take responsibility for the integrity of the data and the accuracy of the data analysis. Study concept and design: BLL, MFS, CIC. Acquisition of data: BLL, MFS, CIC. Analysis and interpretation of data: BLL, MFS, CIC. Drafting of the manuscript: BLL, MFS, CIC. Critical revision of the manuscript for important intellectual content: BLL, MFS, CIC. Administrative, technical, or material support: BLL, MFS, CIC. Study supervision: BLL, MFS, CIC. CIC, BLL and MFS had full access to all the data in the study and take responsibility for the integrity of the data and the accuracy of the data analysis. All authors read and approved the final manuscript.
